# Suramin, an antiparasitic drug, stimulates adipocyte differentiation and promotes adipogenesis

**DOI:** 10.1186/s12944-023-01980-3

**Published:** 2023-12-13

**Authors:** Hanxiao Li, Yingyue Dong, Chunmiao Han, Lisha Xia, Yue Zhang, Tongsheng Chen, Huamin Wang, Guoheng Xu

**Affiliations:** 1 Department of Physiology and Pathophysiology, School of Basic Medical Sciences, Peking University, 100191 Beijing, China; 2 Key Laboratory of Functional and Clinical Translational Medicine, Department of Physiology, Xiamen Medical College, 361023 Xiamen, China; 3 State Key Laboratory of Vascular Homeostasis and Remodeling，Peking University, Beijing, China

**Keywords:** Suramin, Preadipocytes, Preadipocyte differentiation, Adipogenesis, Superficial fascia

## Abstract

**Background:**

Previous studies demonstrated that mast cells with their degranulated component heparin are the major endogenous factors that stimulate preadipocyte differentiation and promote fascial adipogenesis, and this effect is related to the structure of heparin. Regarding the structural and physiological properties of the negatively charged polymers, hexasulfonated suramin, a centuries-old medicine that is still used for treating African trypanosomiasis and onchocerciasis, is assumed to be a heparin-related analog or heparinoid. This investigation aims to elucidate the influence of suramin on the adipogenesis.

**Methods:**

To assess the influence exerted by suramin on adipogenic differentiation of primary white adipocytes in rats, this exploration was conducted both in vitro and in vivo. Moreover, it was attempted to explore the role played by the sulfonic acid groups present in suramin in mediating this adipogenic process.

**Results:**

Suramin demonstrated a dose- and time-dependent propensity to stimulate the adipogenic differentiation of rat preadipocytes isolated from the superficial fascia tissue and from adult adipose tissue. This stimulation was concomitant with a notable upregulation in expression levels of pivotal adipogenic factors as the adipocyte differentiation process unfolded. Intraperitoneal injection of suramin into rats slightly increased adipogenesis in the superficial fascia and in the epididymal and inguinal fat depots. PPADS, NF023, and NF449 are suramin analogs respectively containing 2, 6, and 8 sulfonic acid groups, among which the last two moderately promoted lipid droplet formation and adipocyte differentiation. The number and position of sulfonate groups may be related to the adipogenic effect of suramin.

**Conclusions:**

Suramin emerges as a noteworthy pharmaceutical agent with the unique capability to significantly induce adipocyte differentiation, thereby fostering adipogenesis.

**Supplementary Information:**

The online version contains supplementary material available at 10.1186/s12944-023-01980-3.

## Introduction

Since the mid-1970s, researchers have concentrated on an enduring quest to uncover agents and factors that can either inhibit or promote adipogenesis as the process of fat cell formation. Various chemicals, hormones, and nutrients have been tested. The mixture of insulin, isobutylmethylxanthine (IBMX), and dexamethasone was established [1], and it is still a well-adopted adipogenic cocktail for inducing the differentiation of 3T3-L1 fibroblasts, murine embryonic fibroblasts, and murine stromal preadipocytes in vitro. Furthermore, the mixture of insulin, triiodothyronine, and biotin is advantageous for inducing adipogenic differentiation of freshly isolated rat primary preadipocytes [2, 3]. Since the late 1990s [4], peroxisome proliferator-activated receptor γ (PPARγ), sterol regulatory element-binding protein 1 (SREBP1), and CCAAT/enhancer binding protein α (C/EBPα) have been identified to play central roles in transcriptional regulation of adipogenesis response to hormonal and nutritional signaling. The commonly recognized genes that govern adipocyte adipogenesis at least include SREBP1, C/EBPα/β, and PPARγ, and their targets, such as fatty acid synthase (FASN), hormone-sensitive lipase (HSL) and adipose-triglyceride lipase (ATGL), fatty acid binding proteins (FABPs), and lipid droplet coating protein perilipins. Hormonal, nutritional, and chemical signaling pathways exert influence on the prominent transcription factors. These factors can directly or indirectly modulate the temporal and spatial expression of adipogenic genes, thereby exhibiting inhibitory or stimulatory effects on preadipocyte differentiation, lipid droplet formation, and adipocyte maturation.

Genetic modifications may remarkably enhance adipogenic differentiation. In contrast, except for few known hormones, the therapeutic drugs that can significantly promote adipose differentiation were rarely identified. The antidiabetic thiazolidinedione may be one of the few drugs that can significantly promote adipogenesis both in vitro and in vivo because of its strong action as a PPARγ agonist [5]. Prior investigations conducted in a laboratory have unveiled the presence of abundant preadipocytes within the superficial fascia, capable of differentiating into mature adipocytes and forming a thin adipose layer [6–8]. Mast cells, adipocytes, and newly formed primitive adipose lobules are spatially correlated with each other in superficial fascia [6, 9, 10]. Notably, investigations have identified mast cells, along with their degranulated component heparin, as the principal endogenous factors responsible for stimulating and facilitating fascial adipogenesis, both in vitro and in vivo [11]. Heparin has been found to promote the transcriptional expression of adipogenic genes, including *pparg* and *cebpb*. Protamine is recognized as a polycation that neutralizes the anticoagulant effect of heparin by binding to the anion of heparin. The adipogenic effect of heparin in fascia-derived stromal cells (FSCs) was abrogated by simultaneous incubation of protamine.

Suramin, historically recognized as the trypanocidal drug for combatting African trypanosomas and onchocerciasis since its discovery in 1922 [12], traces its origins back to Paul Ehrlich’s pioneering research, who is mainly regarded as the father of chemotherapy [13, 14]. Suramin is an effective inhibitor of reverse transcriptase and has been investigated as an antiviral agent against HIV [15], Chikungunya virus [16], Ebola virus [17], and recently, SARS-CoV-2 [18]. Suramin has antineoplastic effects [19–21] because of its antagonist actions on various growth factors and cellular proteins and enzymes [22–24]. However, results of clinical phase I-III trials of suramin as an antitumor or antiviral drug have been disappointing because of its numerous reversible toxic effects and low therapeutic efficacy [25].

Structurally, heparin is a highly sulfated glycosaminoglycan, and suramin is a polysulfonated naphthylurea. In the 1980s, the term “heparin-like structure” of suramin was proposed [26]. Regarding the structural and physiologic properties of the negatively charged polymers, suramin is assumed to be a heparin-related analog or heparinoid [26–28]. Due to their polyanionic properties, both heparin and suramin may interact with a variety of proteins and molecules [29, 30]. Numerous heparin-like polysulfated or polysulfonated compounds derived from heparin and suramin also play a wide range of antiviral and antitumor roles. It is interesting to examine whether suramin, like heparin, has a potential effect on adipogenic process. In the present study, the pharmacologic effects of suramin on inducing preadipocyte differentiation and promoting adipogenesis was investigated in this study. It was unveiled that suramin significantly promoted the differentiation of fascial preadipocytes, and this effect was attributable to its polyanionic properties. These discoveries not only shed new light on the multifaceted role of suramin, but also unveil its potential for repurposing as a valuable agent with novel applications.

## Materials and methods

### Materials

Suramin sodium salt was acquired from MedChemExpress (Monmouth Junction, NJ, USA). Protamine was purchased from Sigma-Aldrich (Saint-Louis, MO, USA). PPADS (CAS 149017-66-3) and NF023 (CAS 104869-31-0) were obtained from Aladdin (Shanghai, China) and NF449 (CAS 627034-85-9) was the product of Cayman Chemical Corp. (Ann Arbor, MI, USA).

### Animals

Sprague-Dawley (SD, male) rats were the selected subjects. The animal investigation was carried out following the NIH guide, and the animal protocol was authorized by the IACUC of the Health Science Center, Peking University.

### Isolation and culture of FSCs cells

Male SD rats, with an average weight of approximately 180 g (± 10 g), were used. The experimental protocol adhered to previously established procedures, as outlined in earlier studies [7, 8, 11]. To initiate the experiment, with meticulous precision, it was attempted to gently elevate the layer of superficial fascia situated between the dermal and deep fascial layers at the hindlimb of these rats, employing surgical tweezers. The fascial sheets were then minced with scissors, and they were subjected to digestion process for 2 h. This process involved the addition of 1 mg/mL of type I collagenase and was carried out in a water bath at a temperature of 37 °C while shaking at a speed of 120 rpm. Subsequent to digestion, the mixture underwent filtration through a #100 steel mesh, followed by brief centrifugation at 1000 ×g. The cell pellets were resuspended in a DMEM supplemented with 10% FBS, seeded, and cultured at 37 °C.

### Adipose-derived stromal cells (ASCs)

Male SD rats, with an average weight of approximately 180 g (± 10 g), were used. The epididymal (Epi) and inguinal (Ing) fat tissue of SD rats were excised and minced, then, digested with type I collagenase (0.8 mg/mL) for 0.5 and 2.5 h, respectively. The digestion mixture was initially subjected to sequential filtration through an 80# steel mesh, followed by a finer 400# steel mesh. The filtrate underwent centrifugation at 800 ×g for a duration of 10 min. The cell pellets were resuspended in a DMEM/F12 (1:1) plus 10% FBS, seeded on 24-well plates, and cultured in an incubator at 37 °C.

### Western blot

Cell lysis was prepared in the buffer enriched with the potent Protease & Phosphatase Inhibitor Cocktail, which sourced from Applygen Technologies (Beijing, China). To ensure precise quantification of protein content, BCA protein assay was conducted. An equivalent protein quantity of 15 µg was loaded, subjected to separation through a 10% SDS-PAGE gel. Subsequently, a precise transfer process was employed to relocate them onto nitrocellulose membranes. Membranes underwent an overnight incubation at a constant temperature of 4 °C with primary antibodies, followed by rinsing and probing with horseradish peroxidase-conjugated secondary antibody. Blots were developed using the highly effective ECL reagents provided by Applygen Technologies. Primary antibodies used were anti-perilipin-1 (ab3526, Abcam, Cambridge, UK), anti-ATGL (PA5-17436, Invitrogen, Carlsbad, CA, USA), anti-FASN (ab22759, Abcam), anti-PPARγ (ab272718, Abcam), anti-C/EBPα (2295, Cell Signaling Technology (CST), Danvers, MA, USA), anti-HSL (4107, CST), and anti-β-actin (3700, CST). Images and results were quantified via ImageJ software.

### RNA extraction and qPCR assay

The RNAtrip kit (Applygen Technologies) was employed to extract the total RNA. Subsequently, a quantity of 2 µg of RNA was subjected to the process of reverse transcription into cDNA, and subjected to quantitative real-time PCR analysis. The gene expression levels were normalized to the level of 18 S rRNA, via the comparative Ct method (2^−ΔΔCt^). The primers are accessible in Table 1.

**Table 1:** Primers employed for the purpose of qRT-PCR

Genes	Forward (5’-3’)	Reverse (5’-3’)
**pparg**	CCGAGAAGGAGAAGCTGTTG	TCAGCGGGAAGGACTTTATG
**cebpa**	ATCCCAGAGGGACTGGAGTT	TTTAGCATAGACGCGCACAC
**cebpb**	TACGAGCCCGACTGCCT	GCGAAGAGGTCGGAAAGGAA
**srebp1**	GCACAGCAACCAGAAACTCA	TCATGCCCTCCATAGACACA
**fabp4**	AGCCCAACTTGATCATCAGC	TCCTGTCATCTGGGGTGATT
**leptin**	AGCAGCTGCAAGGTCCAAG	CAGGGGTCTCCAGCACATTT
**lpl**	CCAGCTGGGCCTAACTTTGA	GGAAAGTGCCTCCATTGGGA
**plin1**	CAGCGGAATATGCTGCCAAC	GAAGAAGGGGCTGACTCCAC
**atgl**	TGTGGCCTCATTCCTCCTAC	CAGATGTCACTCTCGCCTGA
**hsl**	GAGACGGAGGACCATTTTGA	AGGAAGGAGTTGAGCCATGA
**fasn**	AAGCCCTTGGGAGTCAAAGT	TAGACGTCAGCAGGTCGATG
**adiponectin**	TGGAATGACAGGAGCGGAAG	ACATGTAAGCGGCTTCTCCG
**18 S rRNA**	CGCTAGAGGTGAAATTCTTG	GGAACTACGACGGTATCTGA

### Cell proliferation assay

Cell Counting Kit-8 (CCK-8), with a remarkable chemical compound, namely WST-8, was used. Inside mitochondria, the dehydrogenase takes part in the reduction of this compound, ultimately yielding a water-soluble formazan product that exudes an orange-yellow hue. Notably, the intensity of this coloration exhibits an inverse relationship with cytotoxicity, rendering it as a reliable indicator of the quantity of viable cells. Fascial preadipocytes were distributed onto 96-well plates (1000 cells/well), and were subsequently treated with different concentrations of suramin (0-140 µM). At precisely 24-hour intervals, the cell culture medium underwent transformation into a fresh high-sugar medium, supplemented with the addition of the CCK-8 solution (TransGen Biotech, Beijing, China) and incubated for 120 min, at 37 °C. Then, a microplate reader was utilized to measure the absorbance at 450 nm. Control samples consisted of cells treated with a drug-free vehicle served as reference.

### Cell viability

Lactate dehydrogenase (LDH) is an enzyme that primarily resides in the cytoplasm. Typically, LDH does not traverse the intact cell membrane under standard physiological conditions. When cellular damage occurs, it prompts an elevation in cell membrane permeability. Consequently, LDH is released into the surrounding medium, serving as a valuable indicator of cellular viability. To assess the vitality of the cells, a LDH activity assay kit was utilized, which was provided by Applygen Technologies. This assay involves the quantification of LDH activity as a measure of cell viability. The process entails measuring absorbance at 440 nm. As a crucial reference, cells treated with a drug-free vehicle were designated as the control group.

### Staining with Oil Red O and Nile Red

Cells underwent fixation utilizing 4% paraformaldehyde. Subsequently, the cells were subjected to a 15-minute staining in a dark environment using a working solution that contained Oil Red O dye. Cells were photographed under an inverted phase-contrast microscope. Oil Red O was extracted utilizing isopropyl alcohol, and the absorbance was measured at 544 nm for quantitative analysis [11]. Alternatively, cells were stained with Nile Red. The cell nucleus was stained with Hoechst 33,258 for 5 min. Nikon Eclipse TE2000-U microscope was utilized to capture immunofluorescent signals.

### Immunofluorescence staining

The cells underwent fixation using 4% paraformaldehyde solution for a duration of 20 min. Subsequently, permeabilization was carried out by treating the cells with 0.2% Triton X-100. 0.8% BSA solution was used to block non-specific binding. Following three rinses with PBS, overnight incubation at 4 °C was performed using the primary antibody anti-perilipin-1 at a dilution of 1:100. This was followed by a 1-hour incubation in the dark at room temperature with Alexa Fluor 488-labelled secondary antibody (1:100).

### Hematoxylin-Eosin staining

Following the decapitation of rats, the Epi and Ing fat pads were isolated and fixed with 4% paraformaldehyde solution for a period of 24 h. The adipose tissue was dehydrated using 30% sucrose solution for 24 h and was then embedded into paraffin (7 μm). To remove the paraffin, the sections were treated with xylene, followed by rehydration in graded ethanol. Finally, The adipocyte area in the sections stained with hematoxylin-eosin was calculated by ImageJ, and the diameter was calculated according to the formula.

### Triglyceride assay

To figure out the extent of fat accumulation, the triglyceride content in the cells was quantified via a Tissue Triglyceride Content Assay kit provided by Applygen Technologies.

### In vivo drug treatments

The present study was conducted utilizing 12 of 3-week-old male SD rats, which were housed under standard animal care conditions. The rat population was stratified into two groups: the control group, consisting of 6 rats, and the suramin group, which also comprised 6 rats. Over the course of 4 weeks, rats belonging to the suramin group were administered intraperitoneal injections of suramin at a dosage of 50 mg/kg on a weekly basis. After the completion of this 4-week period, rats were decapitated, and subsequent experiments were conducted.

### Statistical analysis

The data shown were mean ± standard deviation (STD). Statistical analysis involved using unpaired Student’s t-test, one-way or two-way ANOVA. *P* < 0.05 was considered statistically significant.

## Results

### Cytotoxic effects of suramin on fascial preadipocytes

**Fig. 1:**
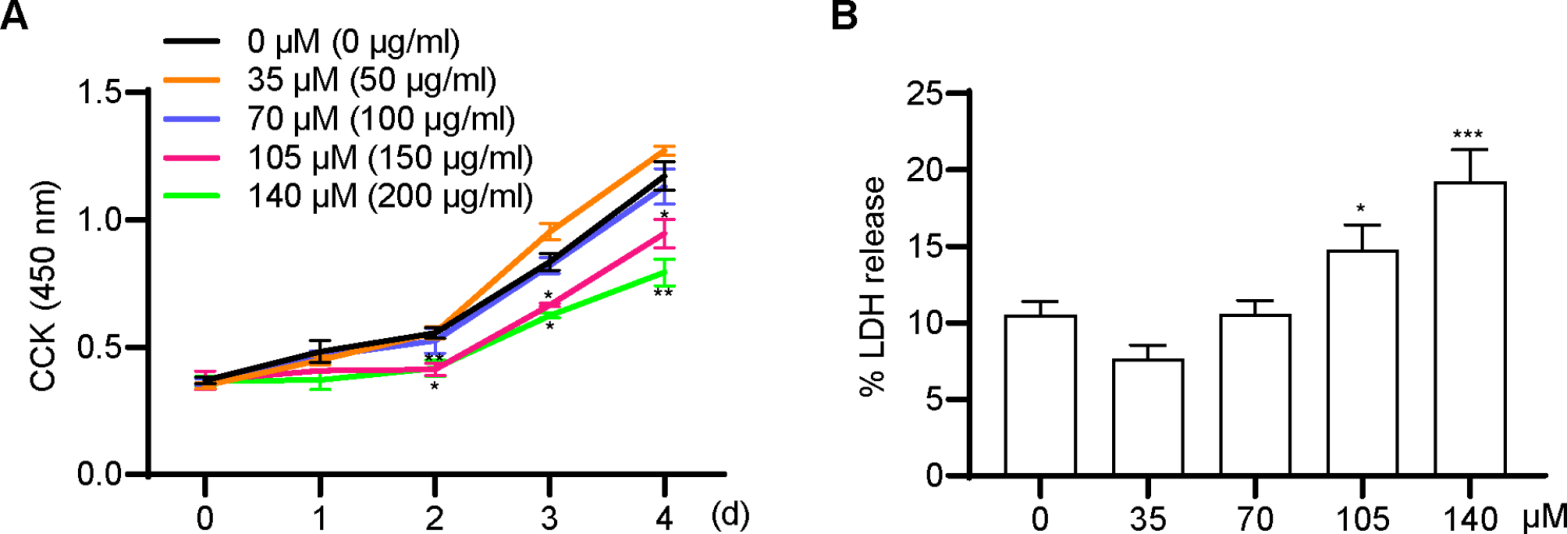
**Effects of suramin on the toxicity of fascial preadipocytes.** Fascial preadipocytes were added to suramin at 35, 70, 105, and 140 µM. (**A**) The CCK-8 assay was used to measure the absorbance at 450 nm to reflect cell viability for 4 days. (**B**) LDH released from fascial cells treated for 5 days with different concentrations (35–140 µM) of suramin. LDH activity in the cell lysate and culture medium was measured at an absorbance of 440 nm via a microplate reader. LDH activity release rate was calculated as follows: (supernatant LDH activity)/(supernatant LDH activity + lysate LDH activity). Data were obtained from three independent experiments and are shown as mean ± STD. Statistical analysis was performed using one-way or two-way ANOVA. *P < 0.05, **P < 0.01, ***P < 0.001

To evaluate suramin’s potential impact on fascial preadipocytes, a comprehensive cytotoxicity assessment was performed by two distinct assays: the CCK-8 assay and the LDH activity assay. CCK-8 assay revealed that suramin at low concentrations of 35 µM (50 µg/mL) and 70 µM (100 µg/mL) showed no cytotoxicity, whereas exhibited a cytotoxic effect by decreasing cell viability at high concentrations of 105 µM (150 µg/mL) and 140 µM (200 µg/mL) (Fig. 1A). Then, LDH release was measured from FSCs treated with suramin for 5 days, which was significantly elevated by adding suramin 105 and 140 µM versus that at 35 and 70 µM (Fig. 1B). According to the CCK-8 and LDH assays, suramin concentration below 70 µM (100 µg/mL) was selected for subsequent experiments.

### Suramin induces adipogenic differentiation of fascial stromal preadipocytes

**Fig. 2:**
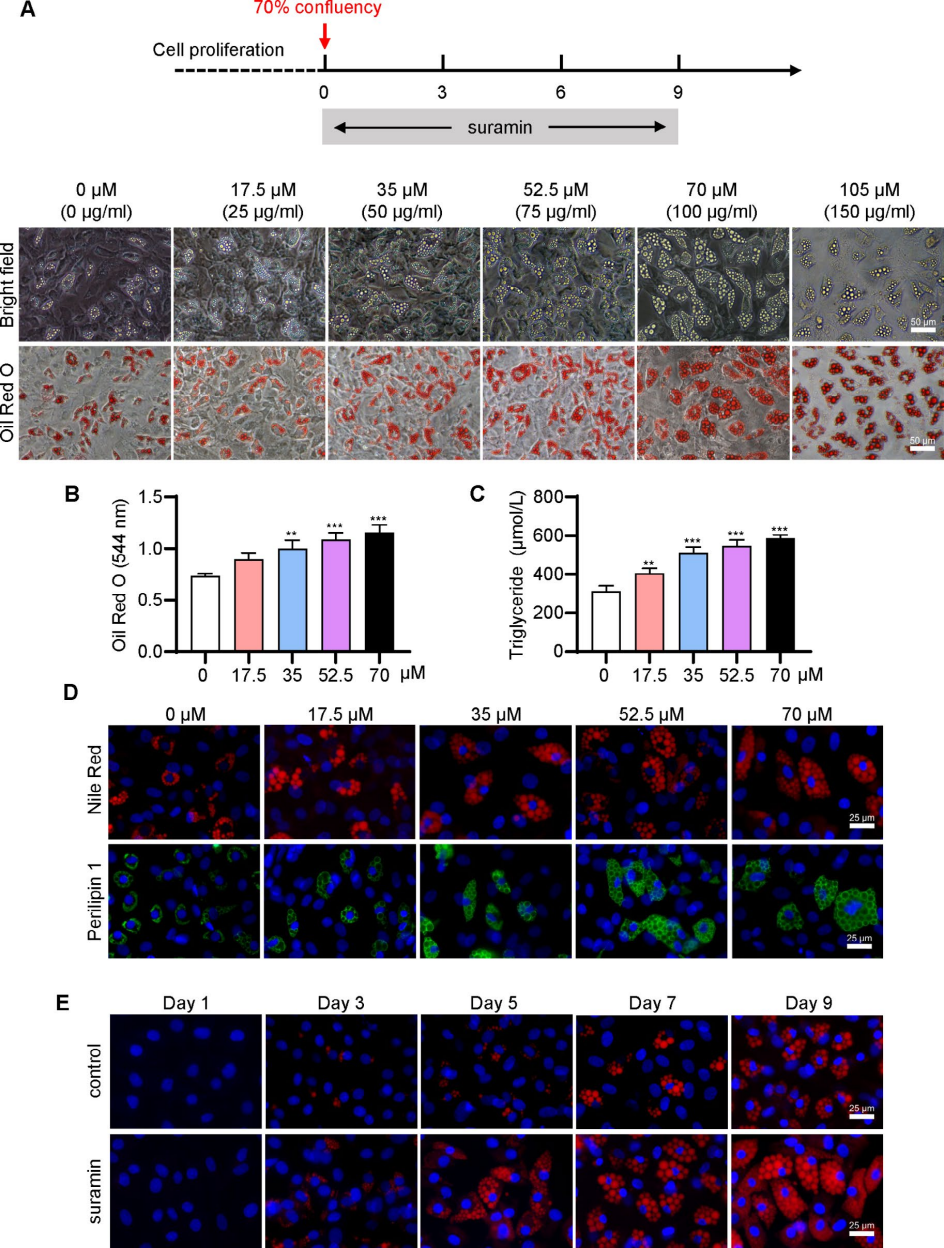
**Suramin treatment promoted adipose differentiation of FSCs** in vitro. (**A**) FSCs grown to 70% confluency received suramin (0, 17.5, 35, 52.5, 70, and 105 µM) in the culture medium. The lipid droplets in cells were stained with Oil Red O and observed by phase-contrast microscopy on day 9. (**B**) Quantitative measurement of Oil Red O within stained lipid droplets on day 10. (**C**) Quantitative analysis of triglyceride content in differentiating FSCs induced by suramin on day 10. (**D**) FSCs were treated with 0–70 µM suramin for 9 days and were then stained with Nile Red or anti-perilipin 1 antibody. (**E**) FSCs were treated with 70 µM suramin and stained with Nile Red on days 1, 3, 5, 7, and 9. Nuclei were visualized by staining with Hoechst 33,258. Data were obtained from three independent experiments and are shown as mean ± STD using one-way ANOVA. **P < 0.01, ***P < 0.001

To figure out the impact of suramin on adipogenesis, it was attempted to isolate and culture subcutaneous superficial FSCs from rats [6–8]. Instead, a spectrum of suramin concentrations was introduced to assess their influence on these preadipocytes. Regarding Oil Red O staining, FSCs treated with 17.5–70 µM suramin developed more lipid droplets versus untreated control (Fig. 2A). Intriguingly, even when exposed to the cytotoxic suramin concentration of 105 µM, the surviving FSCs displayed a robust differentiation capability. This was evident through the proliferation of substantial lipid droplets within the cytoplasm, as depicted in Fig. 2A. Moreover, suramin exhibited a dose-dependent capacity to facilitate the intracellular accumulation of lipid droplets, a phenomenon corroborated by both the Oil Red O staining results (Fig. 2B) and an elevation in cellular triglyceride levels (Fig. 2C). This phenomenon was further validated through Nile Red staining, which unveiled a progressive augmentation in the size and quantity of lipid droplets in response to increasing suramin concentrations (Fig. 2D). Perilipin-1 coats the surface of lipid droplets exclusively in adipocytes. Immunofluorescence staining revealed that suramin dose-dependently increased the ring-like fluorescence signal of perilipin-1 at the surface of lipid droplets within differentiated FSC adipocytes (Fig. 2D). The abovementioned observations were also confirmed by quantification of the fluorescent intensity of Nile Red staining and perilipin-1 staining (Fig. S1A). Treatment with 70 µM suramin gradually increased cytosolic lipid droplets in terms of number and size, on days 3, 5, 7, and 9, as detected by Nile Red staining and quantitative analysis of fluorescent intensity (Fig. 2E and Fig. S1B). These results indicated that suramin stimulated and promoted adipogenic differentiation of FSCs time- and dose-dependently.

### Suramin induces adipogenic differentiation of adult ASCs

**Fig. 3:**
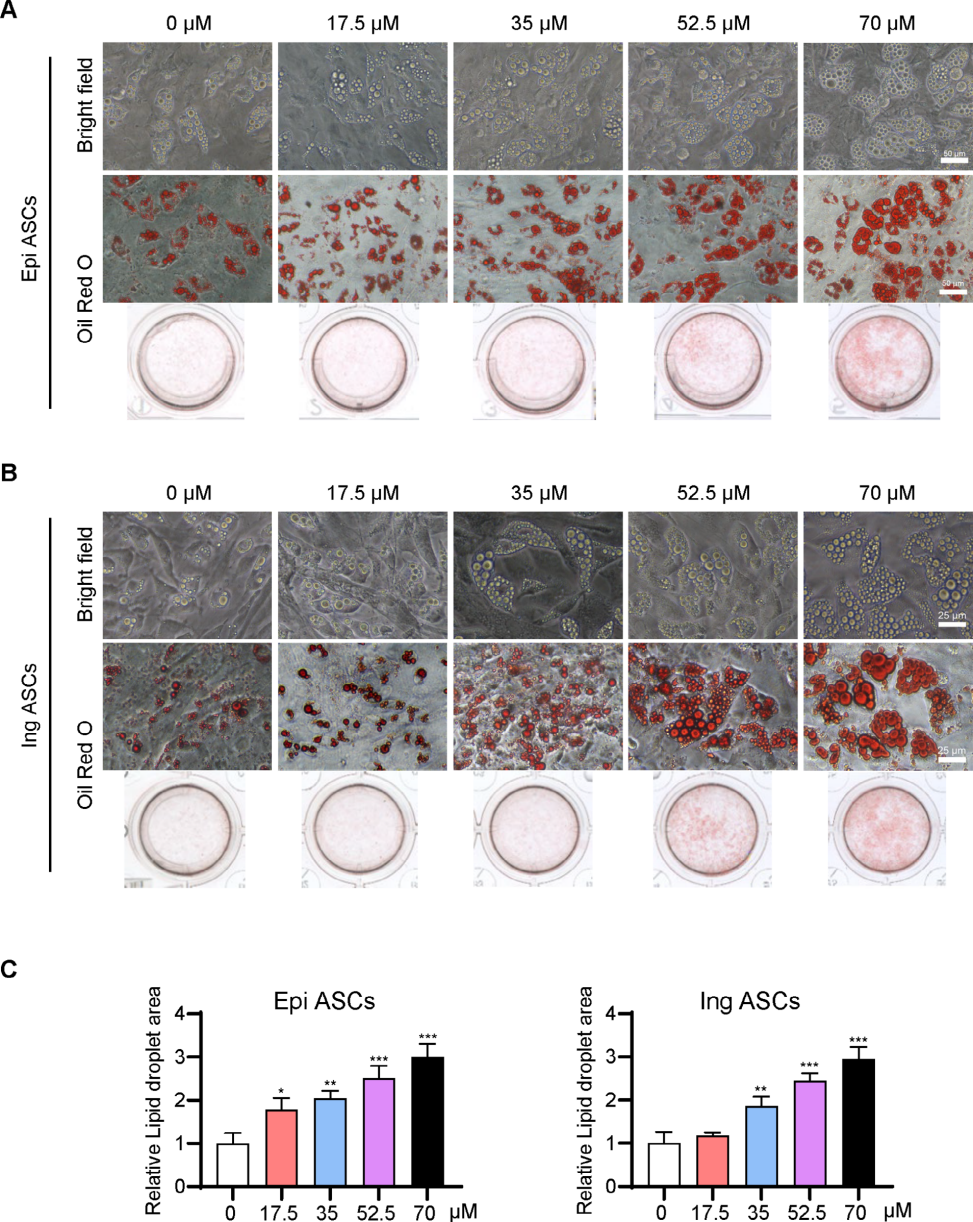
**Suramin promoted adipogenic differentiation of Epi ASCs and Ing ASCs.** Epididymal adipose-derived stromal cells (Epi ASCs) or subcutaneous adipose-derived stromal cells (Ing ASCs) were isolated from rats. When cells grew to 70% confluency, the culture medium was supplemented with suramin (0–70 µM). (**A**) Oil Red O staining showing that suramin could promote adipogenic differentiation of Epi ASCs. (**B**) Oil Red O staining illustrating that suramin could promote adipogenic differentiation of Ing ASCs. The bottom row shows whole cells stained with Oil Red O. (**C**) The lipid droplet area in Fig. 3A and B was measured using ImageJ. Data were obtained from three independent experiments and are shown as mean ± STD from one-way ANOVA. *P < 0.05, **P < 0.01, ***P < 0.001

Next, adult ASCs were isolated from the visceral Epi and subcutaneous Ing fat pads of rats. Preadipocyte differentiation of Epi ASCs and Ing ASCs was induced with suramin at 0, 17.5, 35, 52.5, and 70 µM for 12 and 8 days, respectively. The results revealed that suramin also exhibited a dose-dependent propensity to promote the adipogenic differentiation of both Epi ASCs (Fig. 3A) and Ing ASCs (Fig. 3B) by employing light microscopy and Oil Red O staining. The comparison of the lipid droplet area also confirmed the abovementioned results (Fig. 3C).

### Suramin promotes the expression levels of key adipogenic factors in FSCs

**Fig. 4:**
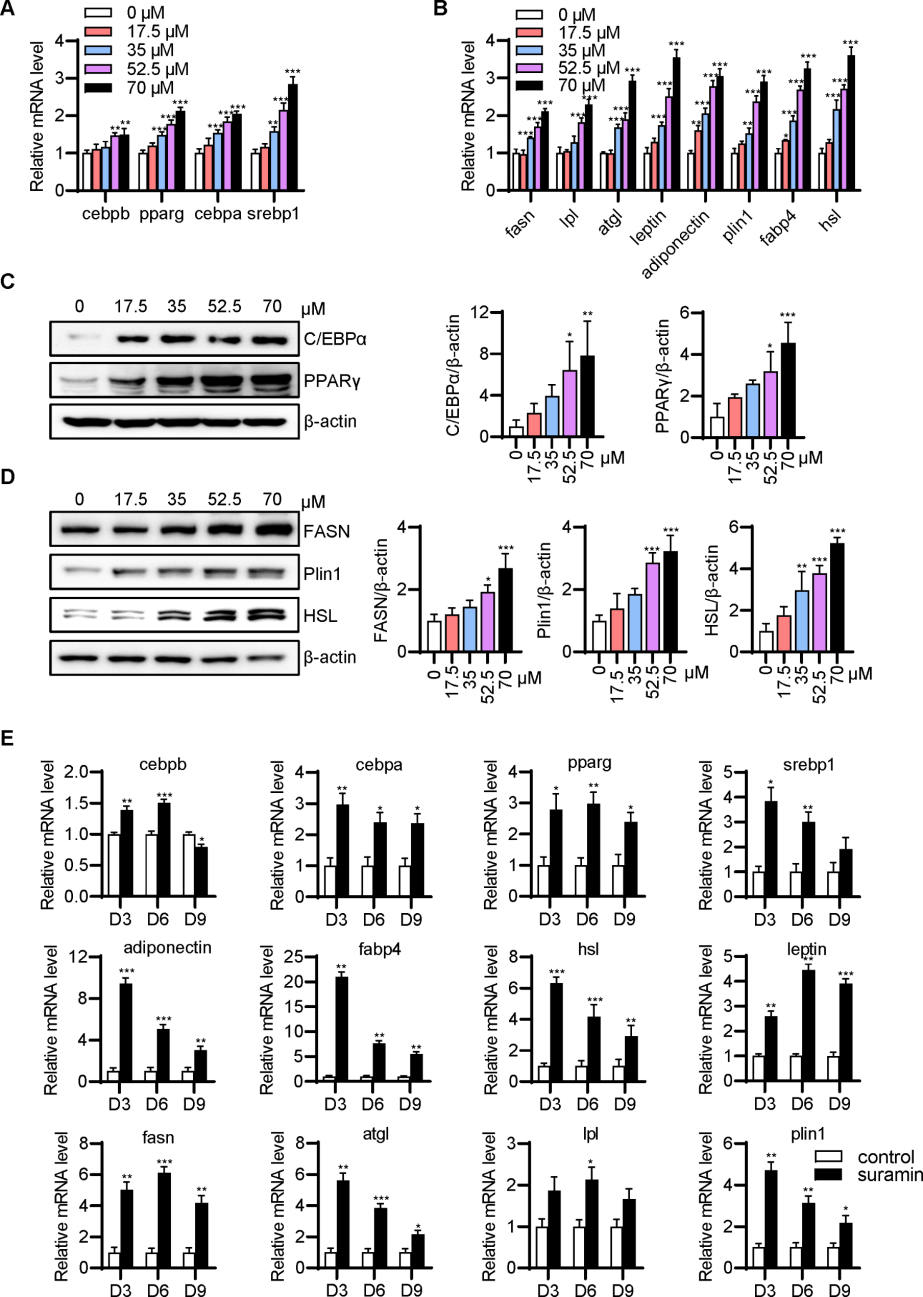
**Suramin promoted the expression of molecules related to adipogenesis in fascia dose- and time-dependently.** (**A**) FSCs were treated with or without suramin for 5 days. Total RNA was extracted, and real-time qPCR was performed with primers specific to adipogenic genes (*cebpb*, *cebpa*, *pparg*, and *srebp1*). (**B**) Real-time qPCR assay of mRNA expression levels of mature adipocyte marker genes on day 5 (*fasn*, *atgl*, *leptin*, *adiponectin*, *plin1*, *fabp4*, *hsl*, and *lpl*). (**C**) FSCs were treated with or without suramin for 5 days, and western blot analysis was performed with antibodies against adipogenic transcription factors (C/EBPα, PPARγ). Band intensity was quantified after normalizing with β-actin. (**D**) Western blot analysis of FASN, Perilipin-1, and HSL on day 9. (**E**) Real-time qPCR for detecting mRNA expression levels of adipogenic genes induced by 70 µM suramin on days 3, 6, and 9, with 18 S rRNA as an internal control. Plin1, perilipin-1. Data were obtained from three independent experiments and are shown as mean ± STD. Statistical analysis was performed using one-way or two-way ANOVA. *P < 0.05, **P < 0.01, ***P < 0.001

The adipocyte differentiation process is controlled by several adipogenic transcription factors and their target genes. Suramin at 17.5, 35, 52.5, and 70 µM dose-dependently upregulated the mRNA expression levels of the key transcriptional factors SREBP1, C/EBPα, C/EBPβ, and PPARγ (Fig. 4A) and consequently increased adipogenic gene expression of FASN, fatty acid binding protein 4 (FABP4, also termed adipocyte-specific protein 2 [aP2]), lipoprotein lipase (LPL), HSL, ATGL, and perilipin-1 (Fig. 4B). The expression levels of mature adipocyte marker genes were identified to be noticeable in FSC adipocytes differentiated by suramin at 52.5 and 70 µM. For example, the *leptin* expression under these two concentrations of suramin was 2.5 times and 3.5 times higher than that in the control group, respectively (Fig. 4B). Consistently, immunoblotting confirmed that suramin dose-dependently increased the protein levels of C/EBPα, PPARγ, FASN, perilipin-1, and HSL in differentiated FSCs (Fig. 4C and D). The expression levels of adipogenic genes during differentiation followed a dynamic process. RT-qPCR findings indicated that the mRNA expression levels of the majority of adipogenic genes were maximized with the utilization of 70 µM suramin on the third day, and their expression levels were then retained high or reduced moderately, whereas remained higher than those in untreated FSCs on days 6 and 9 (Fig. 4E).

### Unique adipogenic differentiation scheme of suramin: dependency on sufficient duration of administration

In general, the time of the addition of adipogenic inducers is important for triggering the preadipocyte differentiation. The most commonly adopted adipogenic cocktail of insulin, IBMX, and dexamethasone are routinely added to confluent 3T3-L1 fibroblasts for incubation for only 2 days [1]. In practice, this 2-day duration of induction sufficiently triggers nearly full differentiation of diverse types of preadipocytes, after being further cultured in the next few days without any adipogenic inducer agents.

**Fig. 5:**
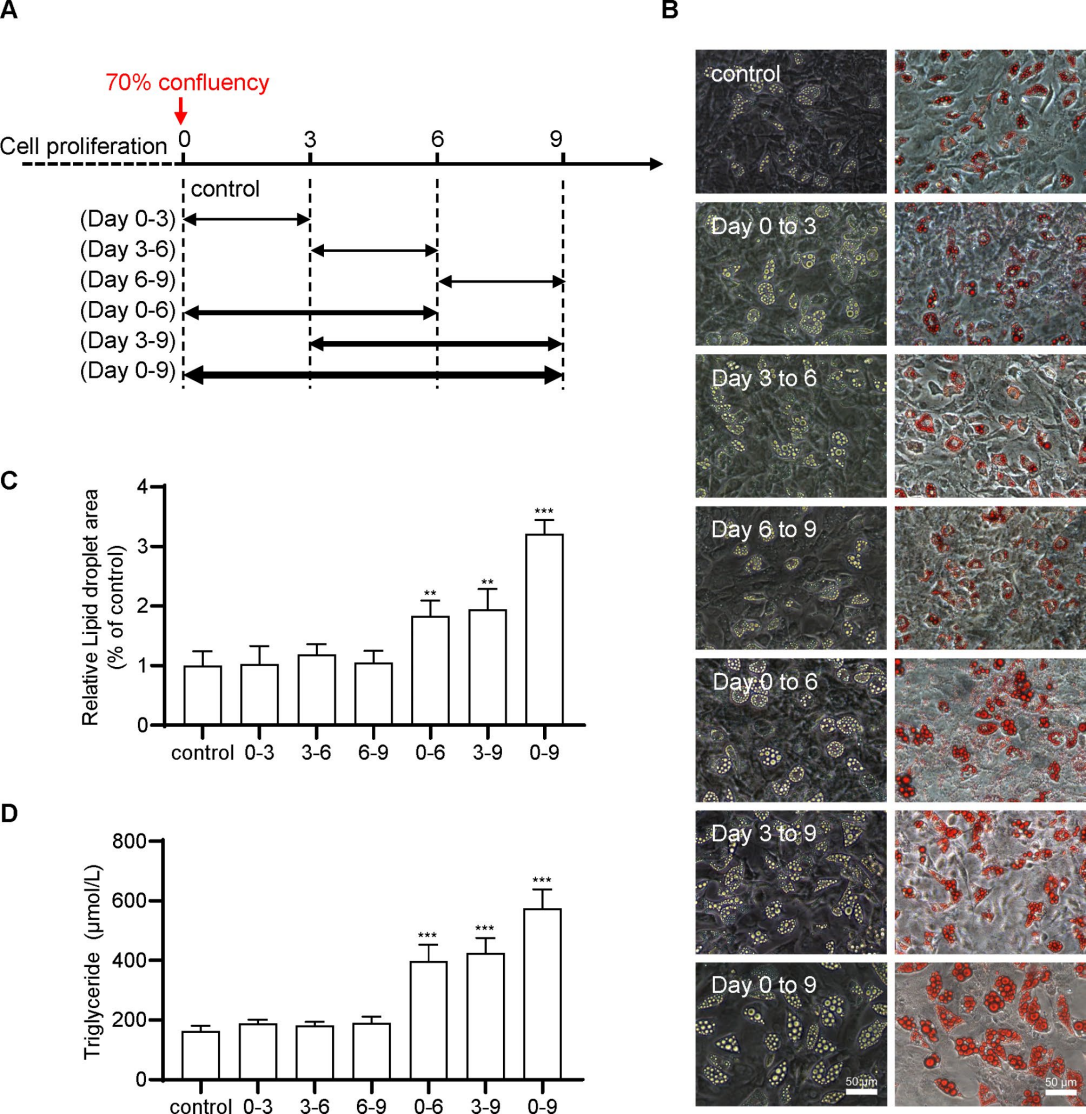
**Adipogenic differentiation effects of suramin depended on the duration of drug administration.** (**A**) The induction scheme for adipose differentiation of FSCs by suramin. The isolated FSCs were plated and cultured to reach 70% confluency. Day 0 was defined as the day before adding suramin. FSCs were incubated with 70 µM suramin from day 0 to 3, day 3 to 6, day 6 to 9, day 0 to 6, day 3 to 9, and day 0 to 9. (**B**) Lipid droplets in adipocytes on day 9 stained with Oil Red O. (**C**) The lipid droplet area in Fig. 5B was measured using ImageJ. (**D**) Quantitative analysis of triglyceride content. Data were obtained from three independent experiments and are shown as mean ± STD. Statistical analysis was performed using one-way ANOVA. **P < 0.01, ***P < 0.001

A duration-dependent administration scheme (Fig. 5A) was used to examine the pharmacological features of suramin on FSC adipogenesis. Regardless of when suramin was added, from day 0 to 3, day 3 to 6, or day 6 to 9, FSCs treated with suramin (70 µM) for only 3 days exhibited comparatively fewer lipid droplets and low adipogenic differentiation (Fig. 5B, C, D). FSCs treated for 6 days with suramin (70 µM), from day 0 to 6 and day 3 to 9, were accompanied by noticeably enhanced lipid droplet accumulation, approximately 2 times that of the control group. The highest level of lipid droplet accumulation was noted in differentiating FSC adipocytes with continuous administration of 70 µM suramin for 9 days, from day 0 to 9 (Fig. 5B, C, D).

### Intraperitoneal injection of suramin slightly promotes adipogenesis in vivo

**Fig. 6:**
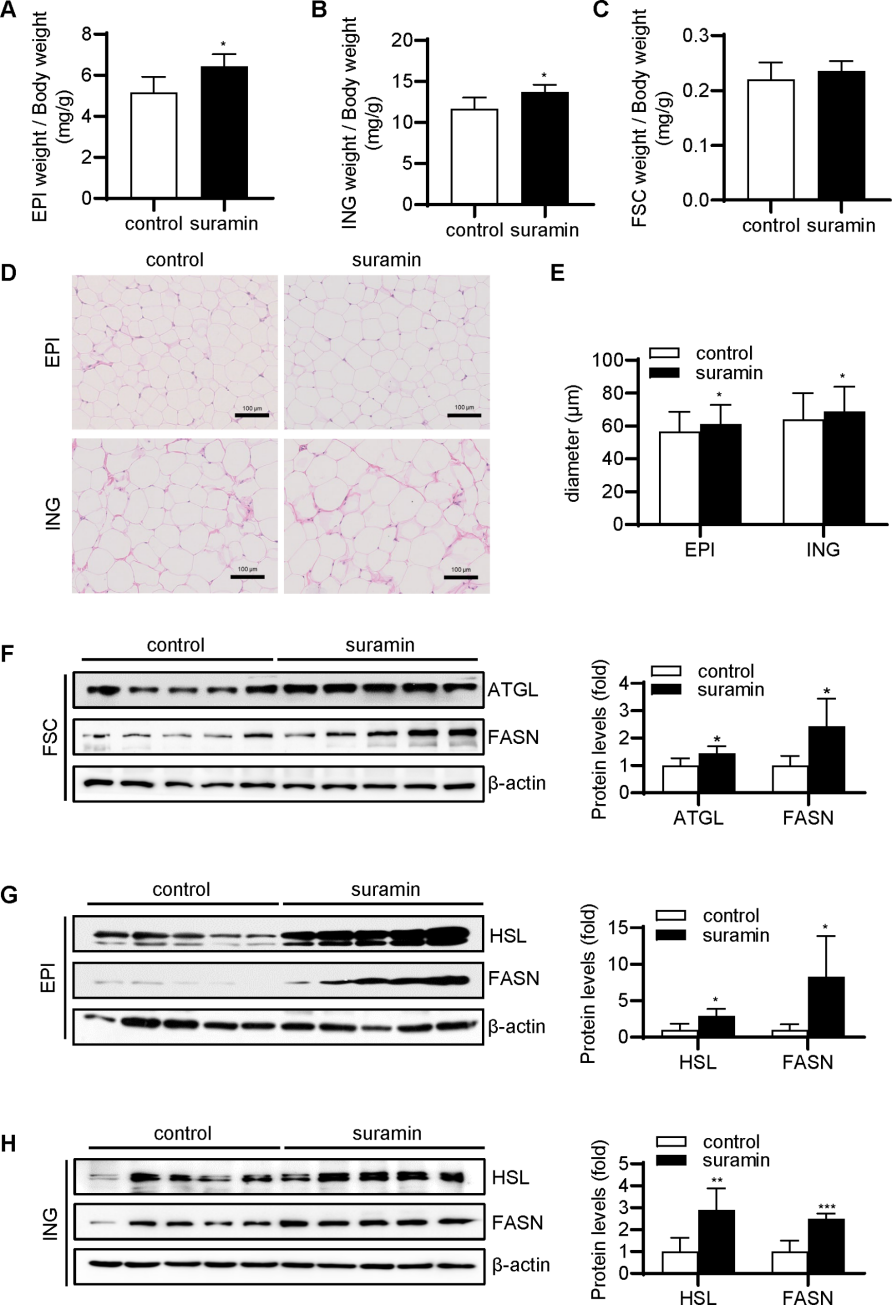
**Effects of intraperitoneal injection of suramin on adipogenesis in rats.** Ratio of (**A**) weight of epididymal (Epi) white adipose tissue to body weight, (**B**) weight of inguinal (Ing) subcutaneous adipose tissue to body weight, and (**C**) superficial fascia fat to body weight. Data are presented as mean ± STD (n = 6). (**D**) Representative images of HE staining of Epi and Ing sections. (**E**) Mean diameter of Epi and Ing adipocytes, n = 6. (**F**) Immunoblot analysis of ATGL and FASN protein expression levels in fascial tissue treated with suramin or saline. (**G**) Western blot analysis of the expression levels of HSL and FASN in the epididymal fat of the control and suramin groups. (**H**) Inguinal subcutaneous fat was collected to detect the expression levels of HSL and FASN. Band intensity was quantified via ImageJ. Data are expressed as mean ± STD and analyzed by two-tailed Student’s t-test. *P < 0.05, **P < 0.01, ***P < 0.001

Next, it was attempted to explore whether long-term administration of suramin could induce adipogenesis *in vivo.* Rats were subjected to intraperitoneal injection of either 50 mg/kg suramin or saline once a week. After 4 weeks, serum biochemistry index was used to assess the drug safety of the selected doses (Table 2). The liver and renal functions of rats were normal and serum lipid levels did not vary with suramin treatment. Suramin and untreated control groups did not differ in weight throughout the experimental period (data not shown). The ratio of weight of Epi (Fig. 6A) or Ing tissue (Fig. 6B) to body weight in the suramin group was 1.2 times that of the control group. Meanwhile, the ratio of superficial fascia fat weight to body weight exhibited an upward trend in the suramin group, whereas the difference from the control group failed to reach statistical significance (Fig. 6C).

**Table 2:** Determination of liver and renal biochemistry indices and blood lipid-related indices in rat serum after intraperitoneal injection of suramin

		control	suramin
**Liver function**	ALT (U/L)	71.55 ± 9.60	81.08 ± 10.26
	AST (U/L)	231.28 ± 33.42	251.23 ± 35.12
	AST/ALT	3.25 ± 0.36	3.11 ± 0.32
	ALP (U/L)	402.43 ± 24.49	466.12 ± 79.33
	TP (g/L)	73.1 ± 2.63	73.02 ± 2.10
	ALB (g/L)	38.4 ± 1.09	38.38 ± 1.34
**Renal function**	CREA (µmol/L)	43.83 ± 7.45	34.97 ± 5.76
	UA (µmol/L)	166.95 ± 51.88	133.25 ± 9.12
	UREA (mmol/L)	9.85 ± 0.89	10.8 ± 0.98
**Blood lipid**	TC (mmol/L)	1.76 ± 0.25	1.57 ± 0.08
	TG (mmol/L)	0.87 ± 0.10	0.94 ± 0.08
	HDL-C (mmol/L)	1.25 ± 0.15	1.19 ± 0.06
	Glycerol (µmol/L)	105.37 ± 14.70	103.87 ± 19.82

Data were expressed as mean ± STD (n = 6). ALT, alanine transaminase; AST, aspartate transaminase; ALP, alkaline phosphatase; TP, total protein; ALB, albumin; CREA, creatinine; UA, uric acid; TC, total cholesterol; TG, triglyceride; HDL-C, high-density lipoprotein cholesterol

Subsequently, Epi and Ing fat pads were embedded into paraffin, and 7-µm sections were prepared. HE staining showed that the adipocytes in the suramin group were slightly larger versus those in the control group (Fig. 6D). The diameter of Epi adipocytes was 56.5 μm in the control group and 61.2 μm in the suramin group. The diameter of Ing subcutaneous adipocytes was 63.9 μm in the control group and 68.7 μm in the suramin group (Fig. 6E). The protein expression levels of adipocyte differentiation markers were risen in superficial fascia and Epi and Ing tissues treated with suramin (Fig. 6F-H). These results suggested that intraperitoneal injection of suramin can slightly promote adipogenesis in rats in vivo.

### Effect of suramin on adipogenesis is related to its sulfonic acid group

**Fig. 7:**
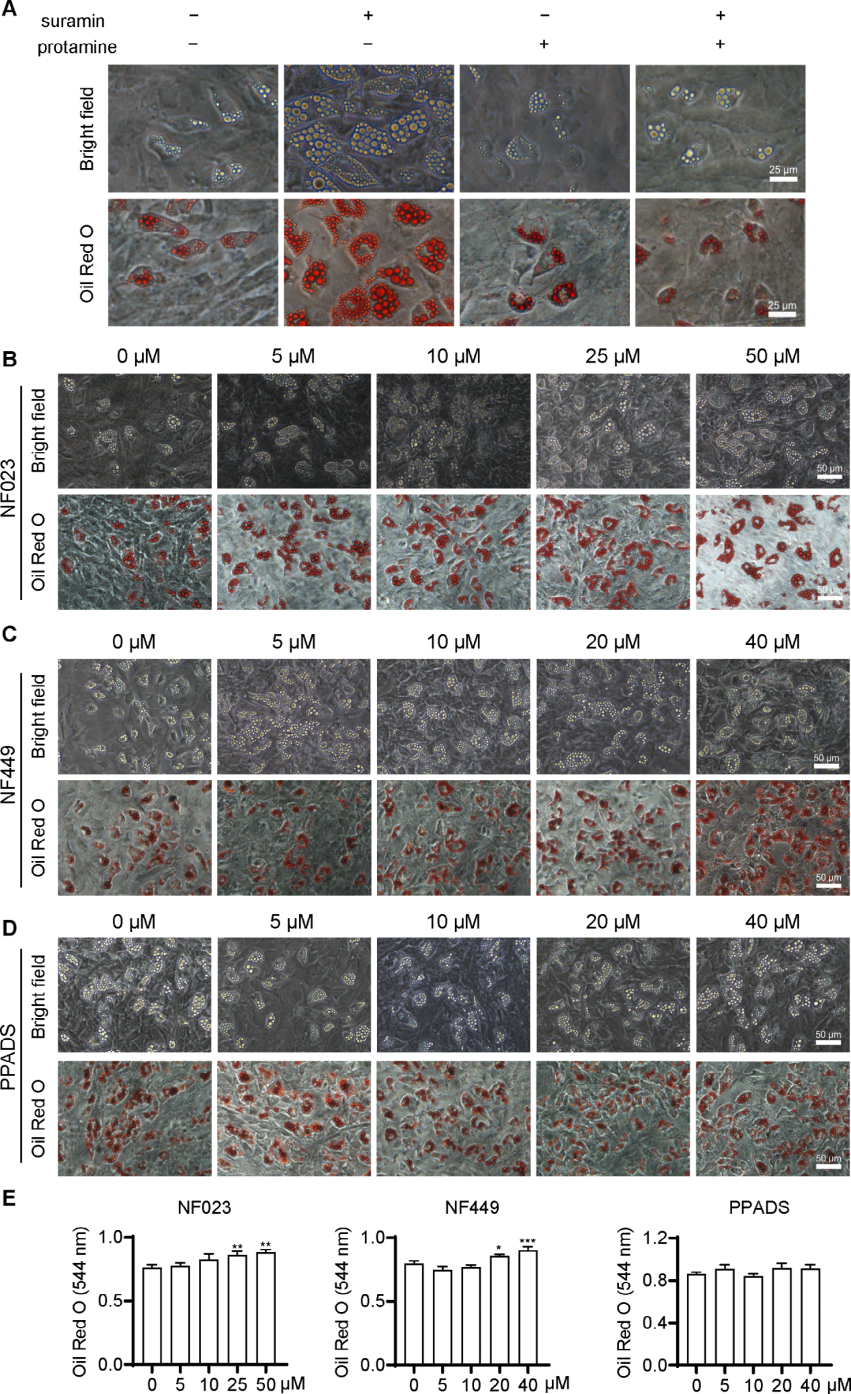
**Effects of suramin on adipogenesis could be related to its sulfonic acid group**. (**A**) Adipogenic effect of suramin with the addition of 50 µg/mL protamine to the culture system to neutralize the anion of suramin (56 µM, equal to 80 µg/mL). Cells were stained with Oil Red O on day 8. (**B**) FSCs were treated with the suramin analog NF023 for 8 days. (**C**) FSCs were treated with the suramin analog NF449 for 8 days. (**D**) FSCs were treated with the suramin analog PPADS for 8 days. (**E**) Quantitative analysis of Oil Red O staining in FSCs treated with the suramin analogs NF023, NF449, and PPADS. Data are expressed as mean ± STD and analyzed by one-way ANOVA. *P < 0.05, **P < 0.01, ***P < 0.001

A previous study revealed that heparin [11] has a similar adipogenic effect as suramin. As both suramin and heparin are polyanionic compounds, it could be speculated that polyanionic groups might be related to the mechanism of adipose differentiation. It was attempted to remove the sulfonic acid group of suramin, and its effect on adipogenic differentiation was studied. Firstly, sulfatase was used to desulfurize suramin, and Fourier transform infrared spectroscopy was then utilized to indicate whether the sulfonic acid group was removed from suramin. The desulfonation of suramin was unsuccessful in the reaction (data not shown) and could be related to the substrate specificity of sulfatase, which is inappropriate for suramin. Secondly, polycationic protamine was incubated with suramin in FSCs. Protamine alone had no influence on FSC adipogenesis, while co-incubation and binding of suramin with protamine that produced flocculent precipitates remarkably weakened the adipogenic differentiation of FSCs (Fig. 7A and Fig. S2). This result indicated that the polyanionic groups of suramin may be an important factor for its differentiation-promoting effect.

Thirdly, it was attempted to indicate whether the number of sulfonic acid groups could determine adipose differentiation. Suramin is a polysulfonated naphthylurea compound containing 6 sulfonic acid groups. Dozens of suramin analogs contain different numbers of sulfonic acid groups, with the position of sulfonate groups varying on the naphthyl ring. Besides, FSCs were incubated with three suramin analogs (the structures are shown in Fig. S3): the analog NF023 contains 6 sulfonic acid groups, NF449 contains 8, and PPADS contains only 2. NF023 and NF449 slightly promoted fascial adipocyte differentiation only in the range of 20–50 µM, which was approximately 1.13 times that of the control group. (Fig. 7B, C). PPADS did not exert any influence on the process of adipogenic differentiation (Fig. 7D). A quantitative assessment of Oil Red O staining results is presented in Fig. 7E.

## Discussion

Suramin, a century-old remedy, remains the pivotal medication for addressing acute human sleeping sickness induced by *Trypanosoma brucei rhodesiense*. It is also employed in managing Trypanosoma evansi infections, particularly in camels [24]. Because of its enigmatic mechanism and diverse repurposing potential applications, suramin has been investigated in the recent century in a great number of animal experiments and clinical trials against various parasitic and viral infections, tumors or as an antitumor chemosensitizer, in multiple myeloma, autism spectrum disorders, and snakebite [24]. The failure of clinical trials with suramin monotherapy is attributable to its numerous toxicities and low therapeutic index [25], which may be related at least in part to its unique pharmacokinetics. In patients, the therapeutic concentration of suramin is 100–350 µg/mL and its half-life is 41–78 days [25].

In this research, it was found that suramin exhibited a robust capacity to stimulate the adipogenic differentiation of preadipocytes obtained from both superficial fascia and adult adipose tissue in vitro. Adipogenic action of suramin appeared at a low concentration, 17.5–35 µM (25–50 µg/mL), and became robust at 52.5 and 70 µM (75 and 100 µg/mL). Suramin at 105 µM (150 µg/mL) exhibited cellular toxicity, whereas still induced numerous large intracellular lipid droplets in surviving differentiating adipocytes. Suramin significantly promoted the expression levels of key adipogenic genes and proteins in differentiated FSCs. Mast cells contain bioactive substances, including heparin, histamine, and 5-hydroxytryptamine. Prior research findings have elucidated that heparin exerts a dose-dependent influence in promoting adipogenic differentiation within preadipocytes originating from the superficial fascia. In contrast, substances (e.g., histamine and 5-hydroxytryptamine), when evaluated within the same context, were found to lack any discernible effect on this differentiation process [11]. Pre-incubation with 5 µg/mL insulin in FSCs for 24 h is required for heparin-induced adipogenic induction. In contrast, suramin alone was sufficient and effective for stimulating strong adipocyte differentiation, without the need for supplementation with any other adipogenic inducers (e.g., insulin). This suggested that the effect of suramin on promoting adipose differentiation was more sensitive and stronger than that of heparin.

Addition of well-known adipogenic cocktail of insulin, IBMX, and dexamethasone to confluent 3T3-L1 cells or FSCs, following incubation for only 2 days, can sufficiently trigger and achieve full adipocyte differentiation [1, 7]. Different from classical adipogenic inducers of insulin, IBMX and dexamethasone, suramin showed a unique pharmacologic action on adipogenic differentiation program. Incubation with suramin for only 3 days exhibited almost no influence on adipose differentiation, regardless of the date administrated on days 0, 3 or 6. Six-day incubation with suramin moderately promoted adipose differentiation, whereas 9-day incubation greatly promoted. Obviously, the stronger differentiation effect of suramin depended on the longer duration of administration rather than a special time point of initial administration. Likely, more than just a simply triggering adipogenic program, the sufficient long duration of action was necessary for suramin achieving full adipocyte differentiation, suggesting that suramin may not act only on classical adipogenic cascades, and other mechanisms may also be involved in the adipogenic action of suramin.

Suramin carries six negatively charged sulfonate groups that may bind to DNA and various proteins and large molecules [24]. Suramin is related closely to polyanionic glycosaminoglycans, heparinoids, and heparin, with similar structural, physiological, and pharmacological properties. A previous study has identified that the adipogenic effects of unfractioned heparin, dalteparin, and enoxaparin were gradually attenuated, which could be correlated with the reduction of their chain lengths of negative glucosamine units [11]. In this study, it was attempted to desulfurize suramin by sulfatase hydrolysis, however, the enzymatic removal of sulfonic acid groups in suramin molecules was unsuccessful, as inspected by Fourier transform infrared spectroscopy. Alternatively, when suramin polyanions were neutralized by polycationic protamine, adipogenic effect of suramin was abolished. Compared with hexasulfonated suramin, PPADS, NF023, and NF449, three suramin analogs [31], contain 2, 6, and 8 sulfonic acid groups. It was revealed that NF023 and NF449 moderately stimulated lipid droplet formation and promoted FSC adipocyte differentiation, while PPADS did not. Furthermore, the number and position of sulfonate groups may be, at least in part, involved in adipogenic differentiation of suramin. These findings provide the information for structure-activity relationship (SAR) on adipogenic action of suramin analogs. However, because sulfatase cannot catalyze desulfonation of suramin analogs, other enzymes or chemical strategies, which can modify the sulfonic groups of suramin, may be advantageous for further addressing this speculation.

Despite a century-long history of therapeutics, the effects of suramin on adiposity and body weight have not yet been figured out. Most clinical trials on suramin have focused on its use in cancer treatment, such as prostate cancer. The main toxic and side effects of suramin include fatigue, anemia, neuropathy, and rash, which may vary depending on the dosage [25, 32]. However, the optimal dosage is currently unclear. These studies have not extensively examined adipose tissue and have rarely reported on the weight of patients or animals. In contrast, thiazolidinedione is known to have potent adipogenic effects both in vivo and in vitro. While the pro-adipogenic effect of insulin is evident at the cellular level, its administration in vivo does not produce the same pro-differentiation effect as observed in vitro. This study found that intraperitoneal injection of suramin into rats slightly increased adipogenesis in epididymal and inguinal adipose tissues, whereas the rat body weight was not altered. However, this effect may not be easily noticeable due to suramin’s ability to bind to numerous proteins and influence various biological processes. Additionally, cell experiments cannot fully replicate the complex extracellular environment. Furthermore, the parasitic and viral infections, tumors, and other diseases treated by suramin are mainly severe chronic consumptive diseases associated with cachexia and weight loss; these pathological effects of the primary diseases may conceal the adipogenic effect of suramin.

In addition, a study published in 2016 found that adipose tissue is a major parasite niche for *Trypanosoma brucei* [33], the pathogen of African sleeping sickness. African *T. brucei* parasites reside in the fat interstitial space between the adipocytes, and they can migrate from adipose tissue to the circulation [33]. Notably, another research indicated that increased adipose tissue might result in a “sponge effect” to sequestrate the parasites, leading to reduction of the parasite load in sensitive organs in high-fat diet-induced obese mice infected with *T. cruzi* [34]. Therefore, it may be worthy to further figure out whether the adipogenic effect of suramin influences the parasite clearance in adipose tissue and the whole body. Adipocyte-like cells and lipid droplets in the model of worm C. elegans have yet been deeply investigated, whether suramin stimulates lipid droplet formation in adipocyte-like cells of African *T. brucei* or other parasites raises another noticeable issue. The present findings provide unique pharmacological clues for exploring these questions in the future.

Previous studies have reported the stimulatory effects on adipogenic differentiation by several sulfonate-containing compounds, such as perfluorobutanesulfonic acid [35], perfluorooctane sulfonate [36], and chlorinated polyfluorinated ether sulfonates [37]. A previous study unveiled that heparin released from mast cells was an endogenous factor for stimulating adipogenesis [11]. Nevertheless, to date, the possible roles of polyanionic groups (sulfonate groups or structures) of these compounds on adipogenic effects have never been examined and discussed. Thus, the pro-adipogenic effects of suramin discussed in this investigation may be valuable for the next research.

### Study strengths and limitations

This study marks the inaugural demonstration of suramin’s notable capacity to substantially enhance adipogenic differentiation of FSCs, particularly evident in in vitro experiments. Notably, suramin does not specifically act on a certain time period of adipose differentiation, suggesting that its adipogenic effect may involve some nonspecific differentiation-promoting mechanism, as well as the classical cascade regulation of transcription factors. This study also preliminarily explored the role of anions of suramin in this process. The limitation is that the mechanism was not deeply explored. Future investigations will greatly benefit from the isolation of suramin’s sulfonic acid group to conduct more intricate experiments. Furthermore, a comprehensive exploration of suramin’s impact on systemic lipid metabolism is worthy of consideration.

## Conclusions

Suramin significantly upregulated the expression levels of lipid-related genes and thus promoted fascial adipocyte differentiation and lipid accumulation. This effect could be attributable to its polyanionic properties. This investigation expanded the understanding of the pharmacological effects of the classic antiparasitic drug suramin. Since adipose tissue severs as a ‘sponge’ for the parasite, this suggests that the lipogenic effects of suramin may affect the clearance of parasites from patients’ adipose tissue.

### Electronic supplementary material

Below is the link to the electronic supplementary material.

Supplementary Material 1

## Data Availability

Data can be made accessible upon request.

## Abbreviations

ATGL: Adipose triglyceride lipase
ASC: Adipose-derived stromal cells
C/EBP: CCAAT-enhancer-binding proteins
Epi: Epididymal
FABP4: Fatty acid binding protein 4
FASN: Fatty acid synthase
FSC: Fascia-derived stromal cell
HSL: Hormone-sensitive lipase
Ing: Inguinal
LPL: Lipoprotein lipase
Plin1: Perilipin 1
PPARγ: Peroxisome proliferator-activated receptorγ
SREBP1: Sterol regulatory element binding transcription factor 1
