# Supplementary material for: Suramin, an antiparasitic drug, stimulates adipocyte differentiation and promotes adipogenesis
Source: Lipids Health Dis. 2023 Dec 13;22:222. doi: 10.1186/s12944-023-01980-3 (PMC10717495; doi:10.1186/s12944-023-01980-3)
Supplement: Supplementary file 1 — Supplementary Material 1 [file 12944_2023_1980_MOESM1_ESM.doc]

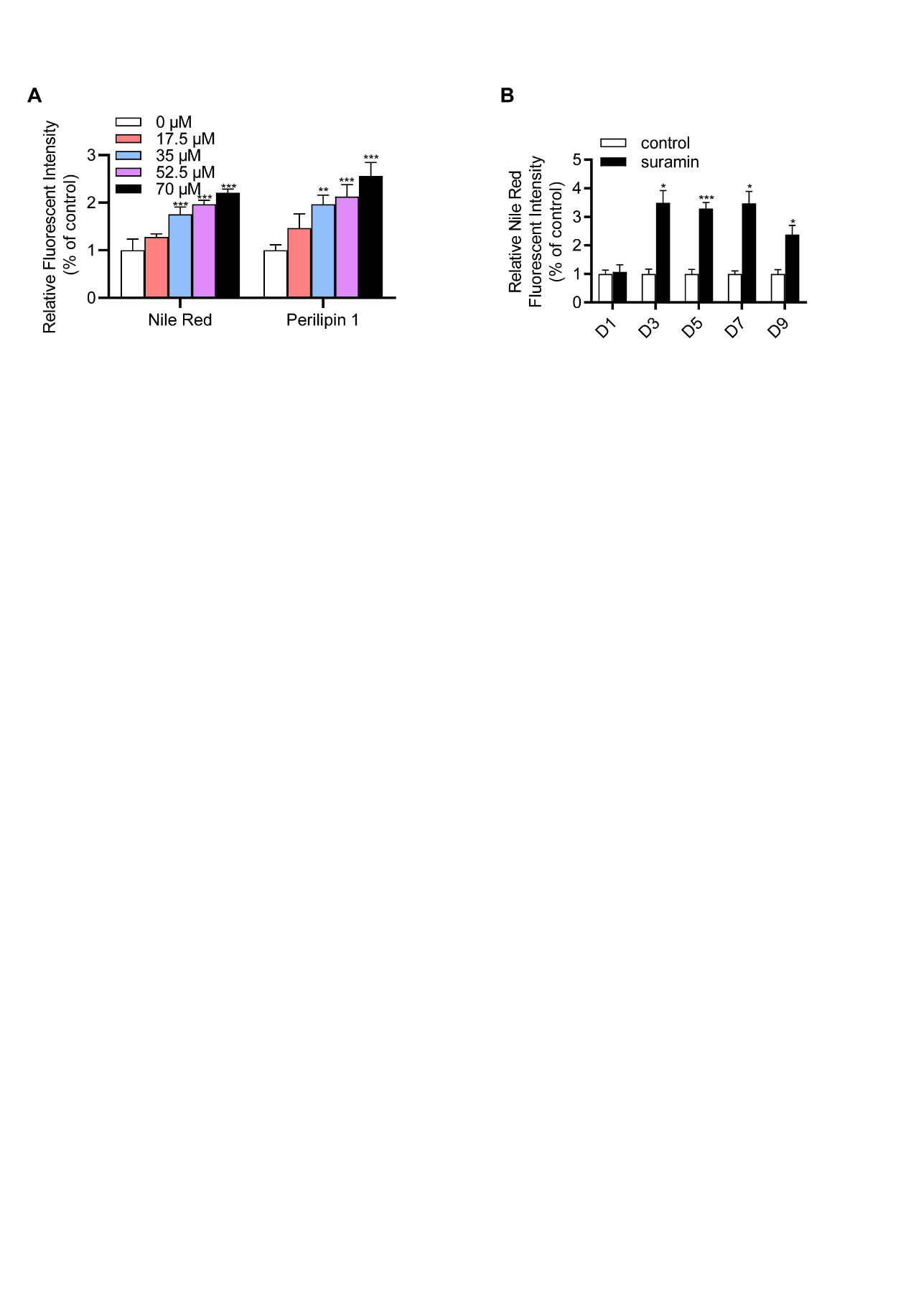


**Figure S1. Suramin promoted adipogenic differentiation of FSCs time- and dose-dependently.** (A) Quantitative analysis of fluorescent intensity in Fig. 2D. (B) Quantitative analysis of Nile Red fluorescent intensity in Fig. 2E. The control group was considered as 1. Data were obtained from three independent experiments and are shown as mean ± STD. Statistical analysis was performed using one-way or two-way ANOVA.**P* < 0.05, ***P* < 0.01, ****P* < 0.001.


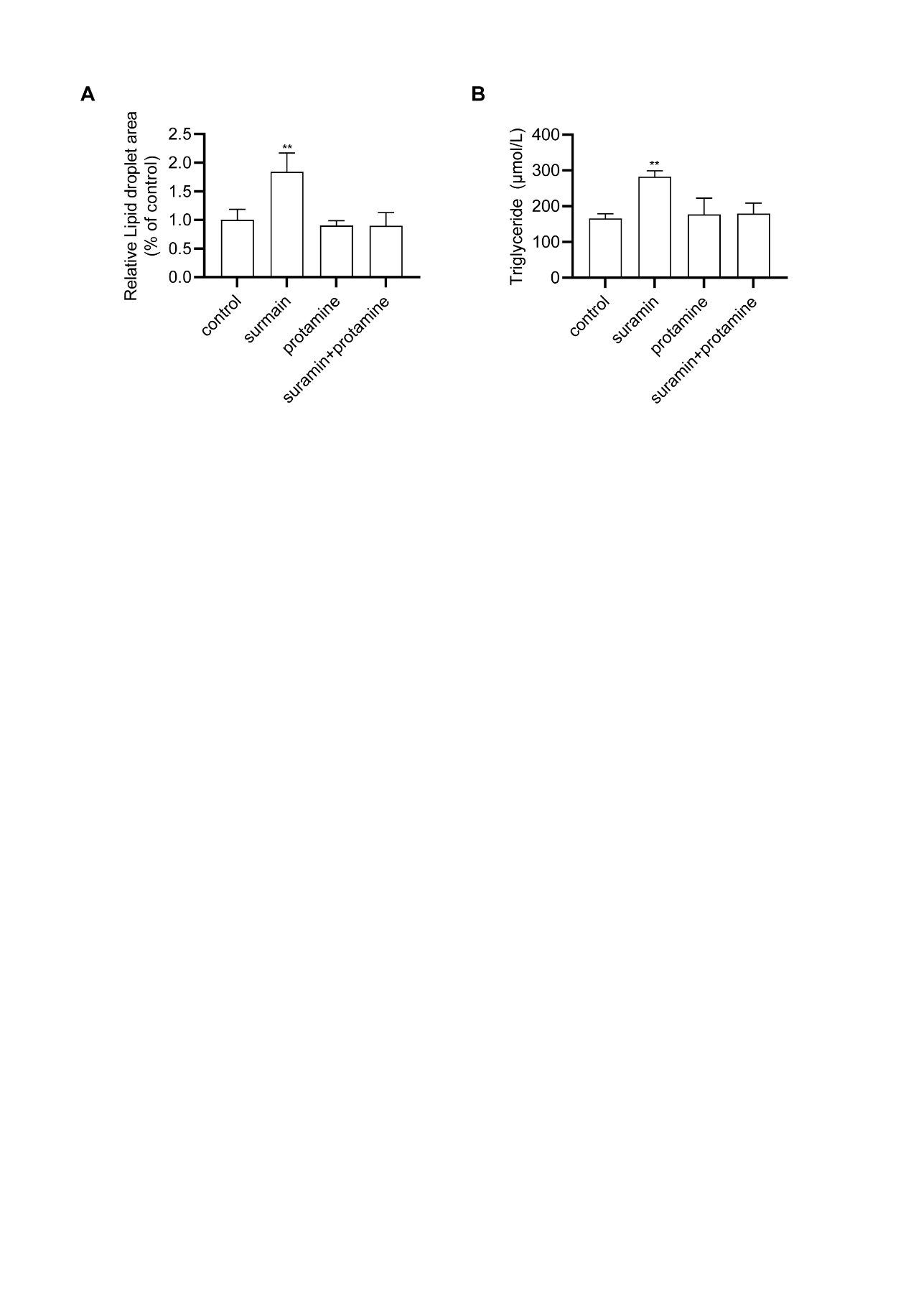


**Figure S2. Addition of protamine abolished the adipogenic effect of suramin.** (A) Lipid droplet area in Fig.7 A was measured using ImageJ. (B) Quantitative analysis of triglyceride content in differentiating FSCs induced by adding suramin and protamine alone or simultaneously on day 8. Data were obtained from three independent experiments and are shown as mean ± STD. Statistical analysis was performed using one-way ANOVA. ***P* < 0.01.


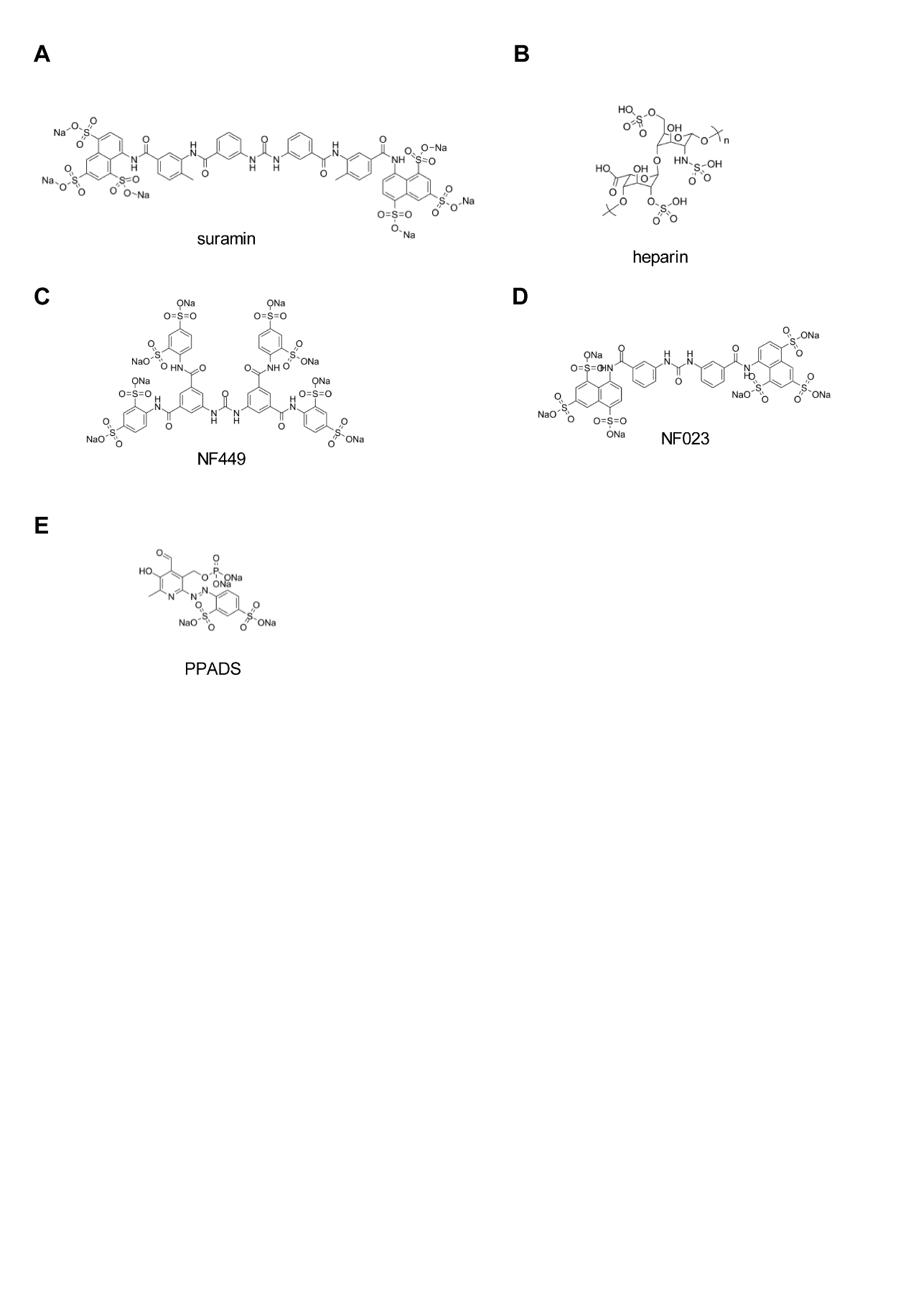


**Figure S3. The chemical structure of suramin and its analogs (suramin, heparin, NF023, NF449, and PPADS).**
